# Supplementary material for: All-printed stretchable corneal sensor on soft contact lenses for noninvasive and painless ocular electrodiagnosis
Source: Nat Commun. 2021 Mar 9;12:1544. doi: 10.1038/s41467-021-21916-8 (PMC7943761; doi:10.1038/s41467-021-21916-8)
Supplement: Supplementary file 1 — Supplementary Information [file 41467_2021_21916_MOESM1_ESM.docx]

**Supplementary Information**

All-Printed Stretchable Corneal Sensor on Soft Contact Lenses for Noninvasive and Painless Ocular Electrodiagnosis

Kyunghun Kim^1†^, Ho Joong Kim^2†^, Haozhe Zhang^3†^, Woohyun Park^4^, Dawn Meyer^5^, Min Ku Kim^1^, Bongjoong Kim^4^, Heun Park^1^, Baoxing Xu^3*^, Pete Kollbaum^5*^, Bryan W. Boudouris^2,6*^, Chi Hwan Lee^1,4,7*^

^1^Weldon School of Biomedical Engineering, Purdue University, West Lafayette, IN 47907, USA. ^2^Charles D. Davidson School of Chemical Engineering, Purdue University, West Lafayette, IN 47907, USA. ^3^Department of Mechanical and Aerospace Engineering, University of Virginia, Charlottesville, VA 22903, USA. ^4^School of Mechanical Engineering, Purdue University, West Lafayette, IN 47907, USA. ^5^School of Optometry, Indiana University, Bloomington, IN 47401, USA. ^6^Department of Chemistry, Purdue University, West Lafayette, IN 47907, USA. ^7^School of Materials Engineering, Purdue University, West Lafayette, IN 47907, USA. ^†^These authors contributed equally: Kyunghun Kim, Ho Joong Kim, Haozhe Zhang. *Correspondence to: bx4c@virginia.edu (B.X.); kollbaum@indiana.edu (P.K.); boudouris@purdue.edu (B.W.B.); lee2270@purdue.edu (C.H.L)

**
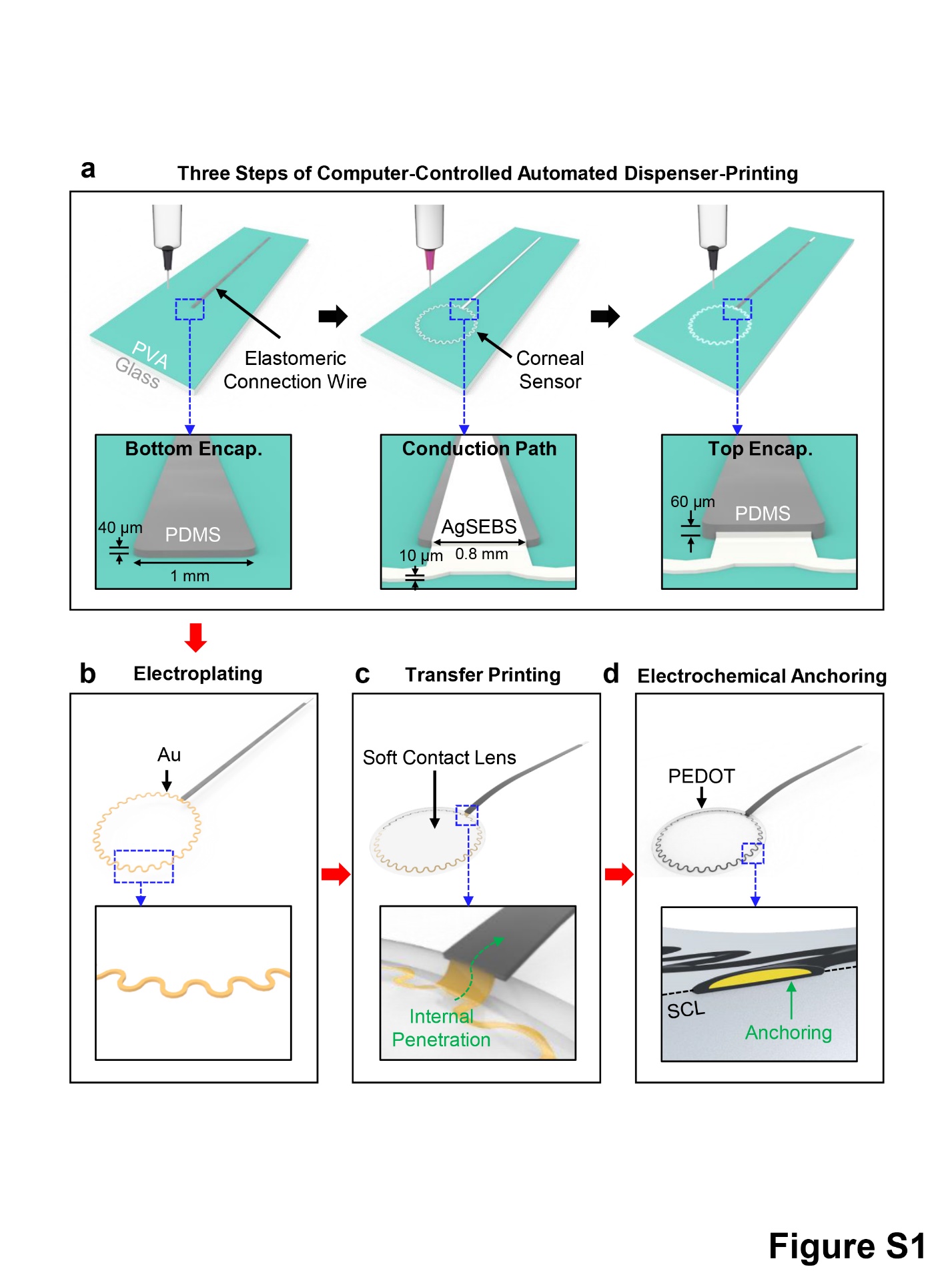
**

**Supplementary Figure S1. Schematic illustration of basic procedures for fabricating the corneal sensor. a**, A series of computer-controlled automated dispenser-printing processes for the fabrication of the corneal sensor on a temporary glass substrate coated with a water-soluble PVA layer. The bottom magnified images show the dimensions of each printed layer. **b**, Electroplating process of the conduction path (i.e., AgSEBS layer) with Au. The bottom magnified image highlights the change of color to gold after the electroplating process. **c**, Transfer printing process of the entire structure from the temporary glass substrate to the inner surface of a SCL. The bottom magnified image highlights the connection wire inserted out through the SCL for seamless integration, **d**, Electrochemical polymerization of EDOT to form a PEDOT layer over the corneal sensor. The bottom magnified image highlights the monolithic anchoring of the corneal sensor to the SCL.


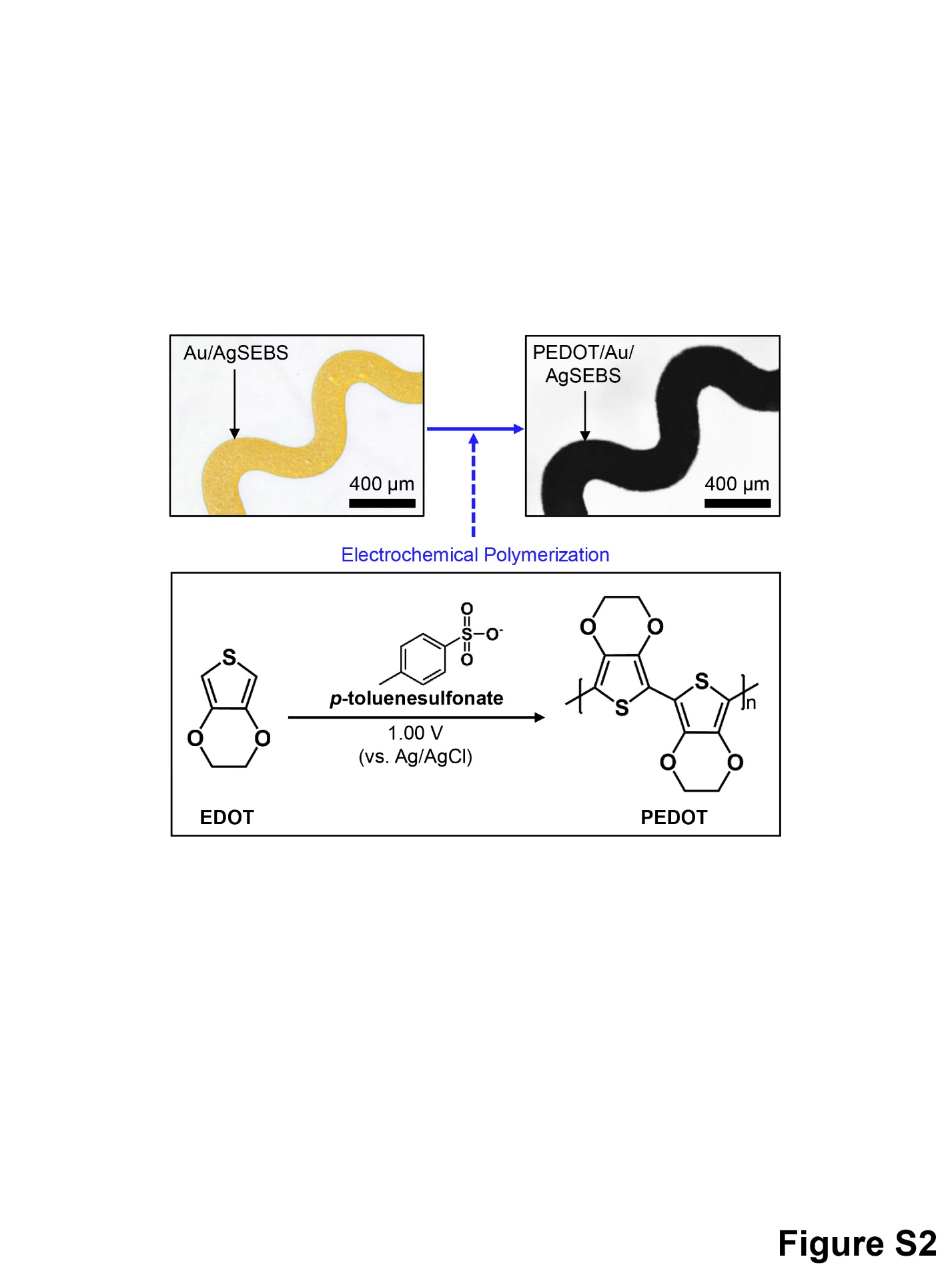


**Supplementary Figure S2. Electrochemical polymerization of PEDOT.** Enlarged microscope images of the corneal sensor before (top left image) and after (top right image) the electrochemical polymerization of PEDOT, with a scheme of the electrochemical polymerization process (bottom image).

**
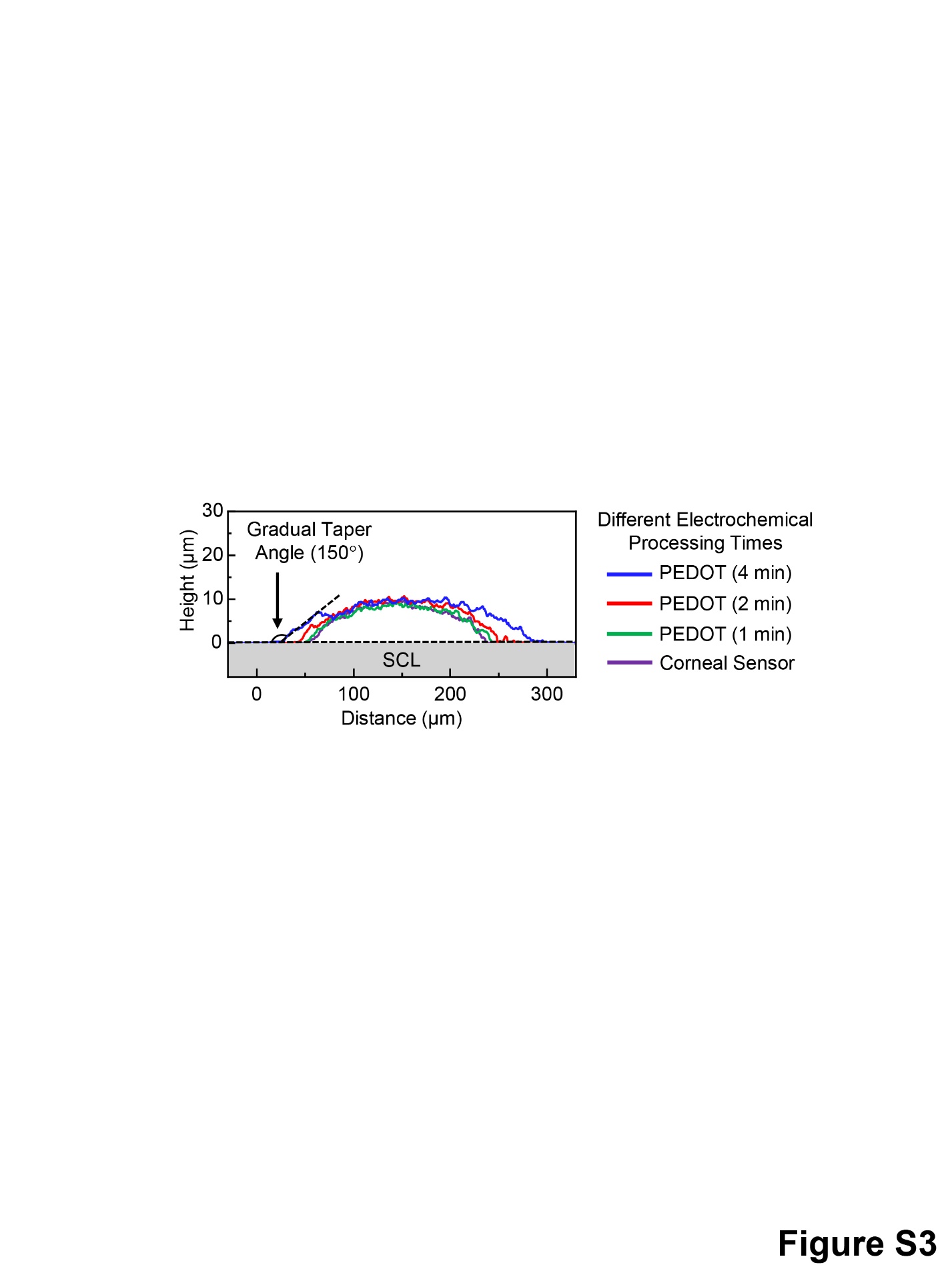
**

**Supplementary Figure S3. Characterization of the electrochemically-grown PEDOT layer.** Surface topology of the corneal sensor with a PEDOT layer prepared at different electrochemical processing times.

**
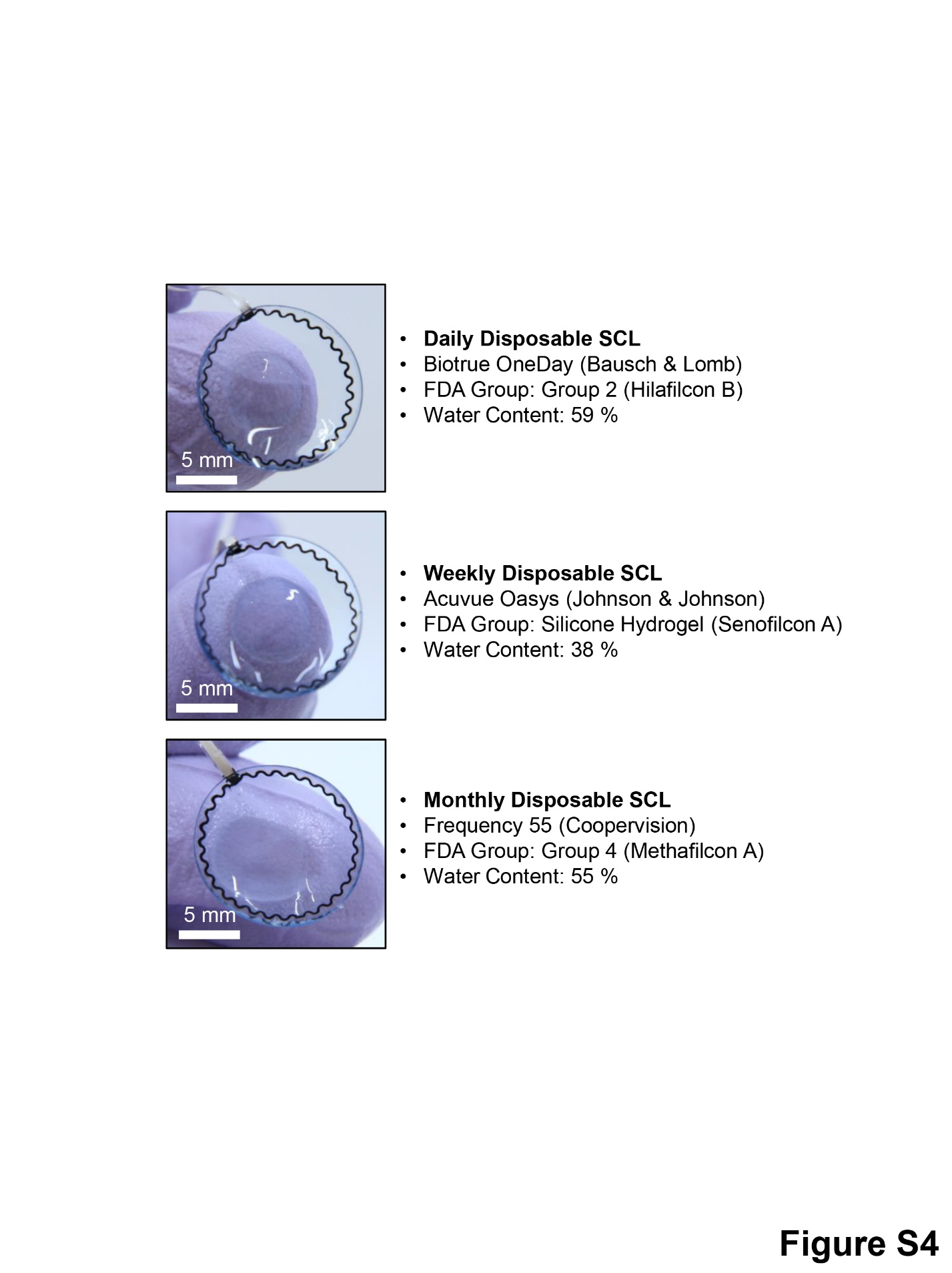
**

**Supplementary Figure S4. Applicability to various types of commercially-available disposable SCLs.** Photographs of the corneal sensor applied to daily, weekly, and monthly disposable SCLs from the top. The commercial name, water content, and classification by the U.S. Food and Drug Administration (FDA) of each SCL are denoted.

**
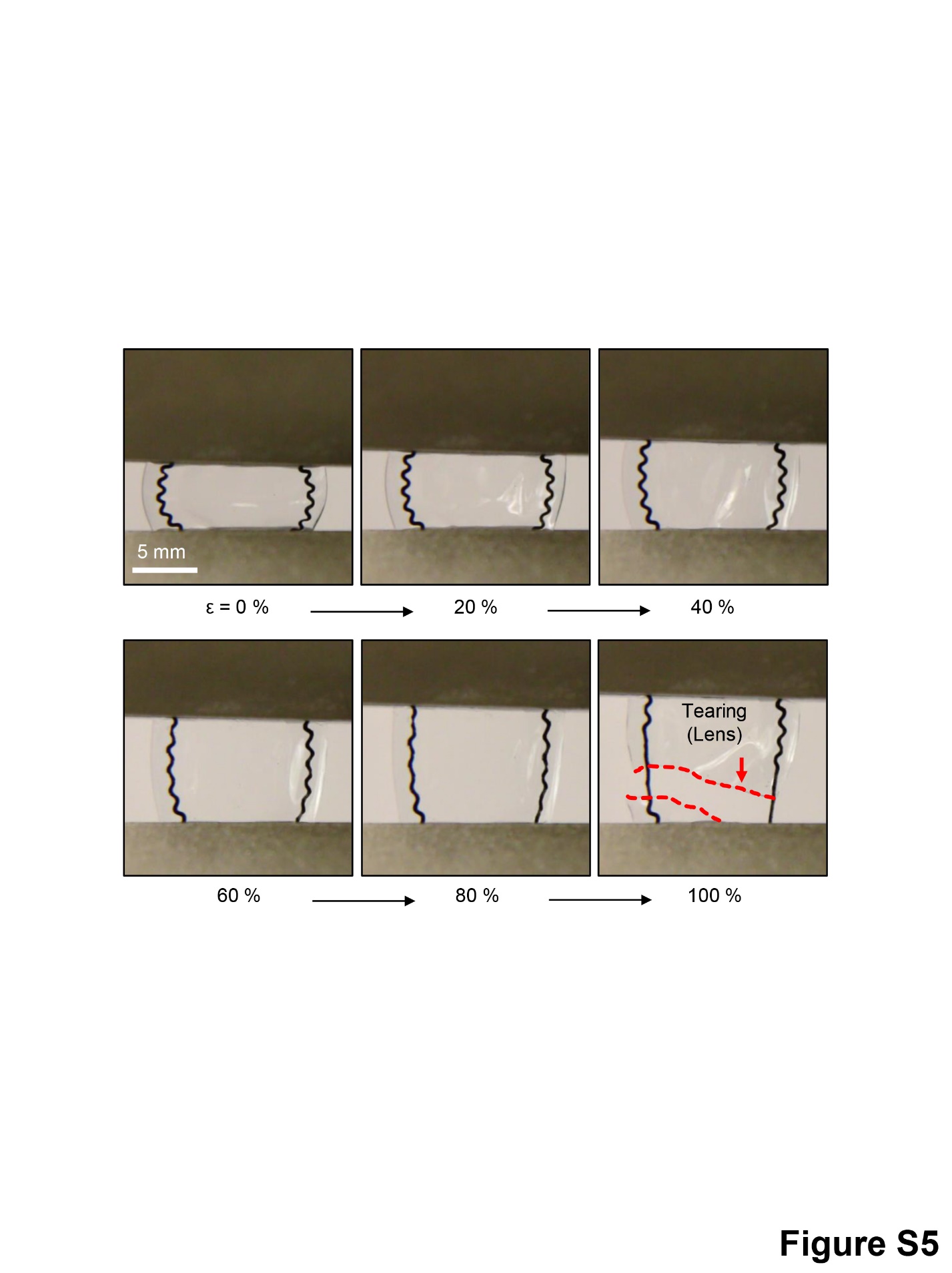
**

**Supplementary Figure S5.** **Stretching of the corneal sensor.** A series of photographs for the corneal sensor under stretching until it reaches the failure point.

**
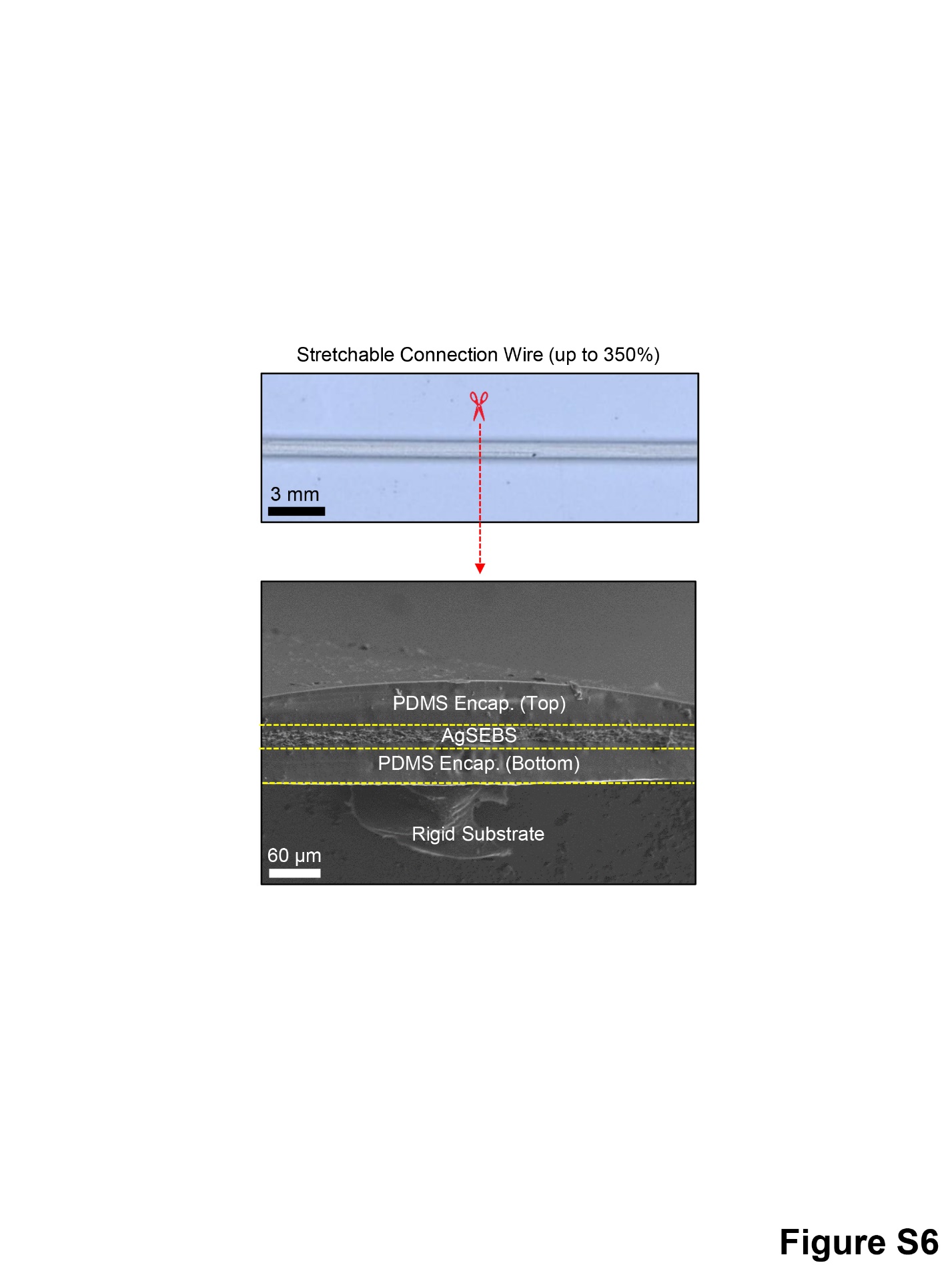
**

**Supplementary Figure S6. Layout of the connection wire.** Top-view photograph (top inset) and cross-sectional scanning electron microscope (SEM) image (bottom inset) of the connection wire.

**
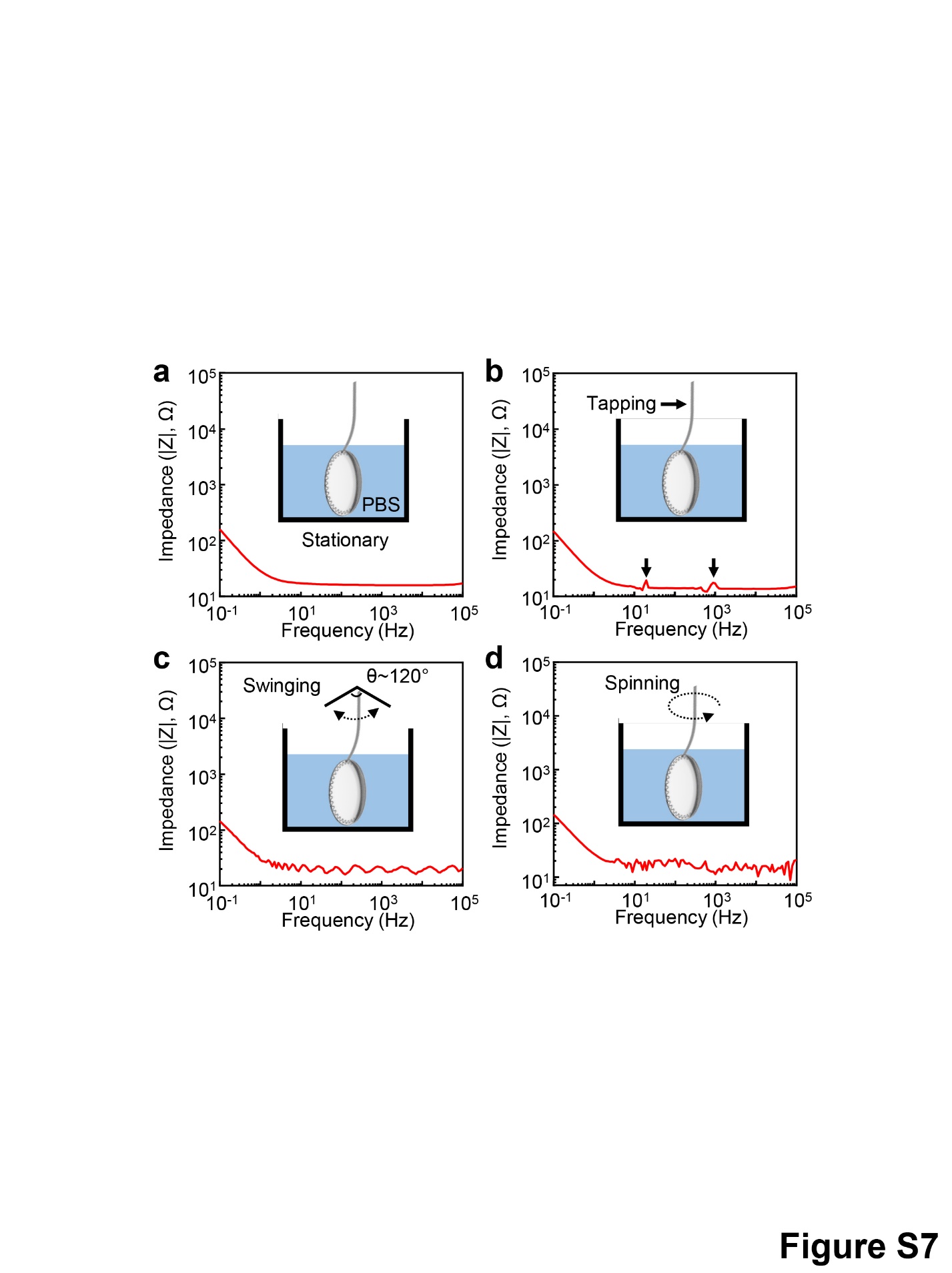
**

**Supplementary Figure S7.** **Electrochemical impedance against various conditions.** Electrochemical impedance of the corneal sensor against tapping, swinging, and spinning of the connection wire, as compared to that under stationary condition.

**
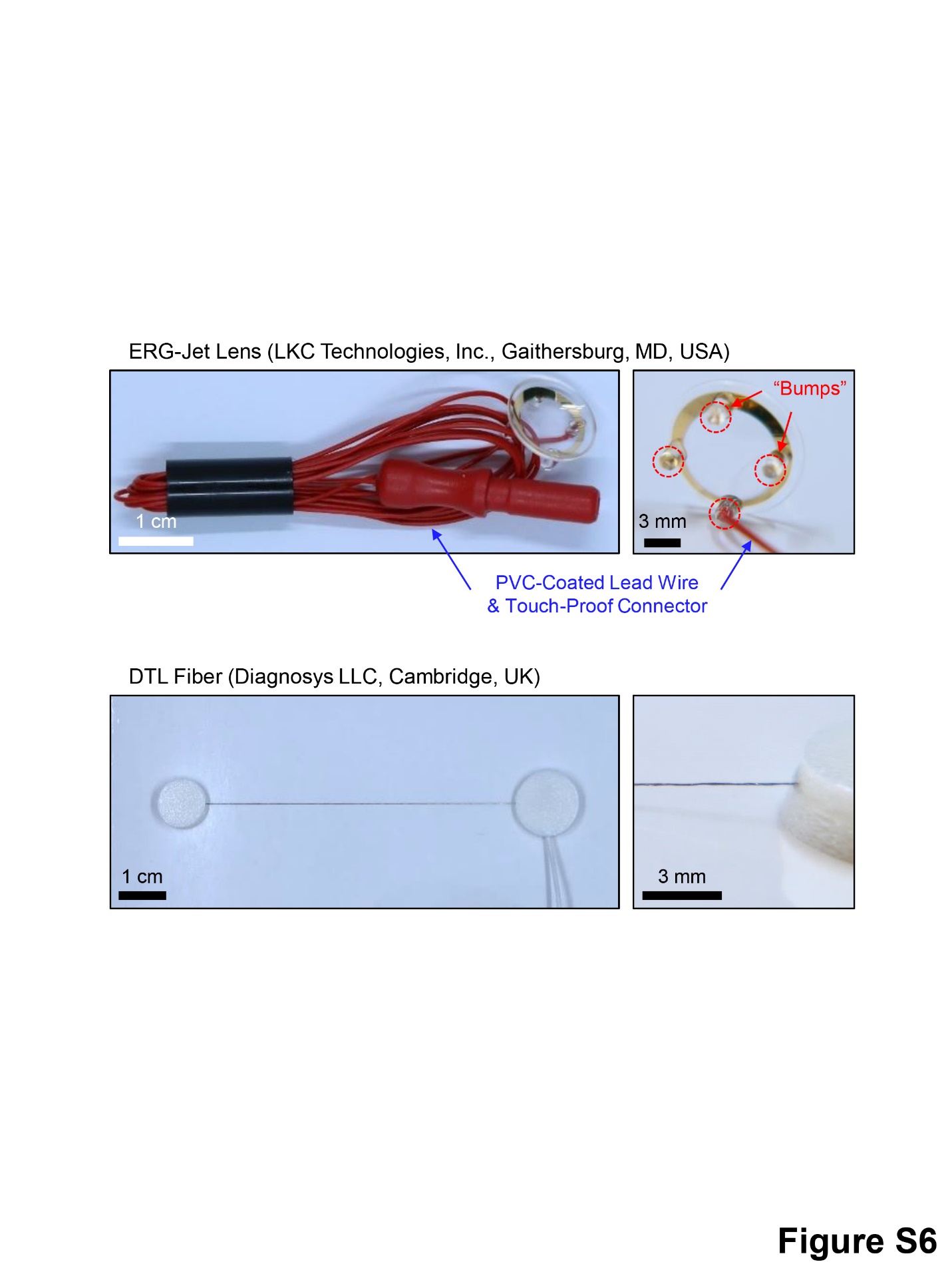
**

**Supplementary Figure S8. Current clinical standards for ERG recording.** Photographs of the ERG-Jet lens (top images) and the DTL fiber (bottom images). The vendors of these devices are noted above each photograph.

**
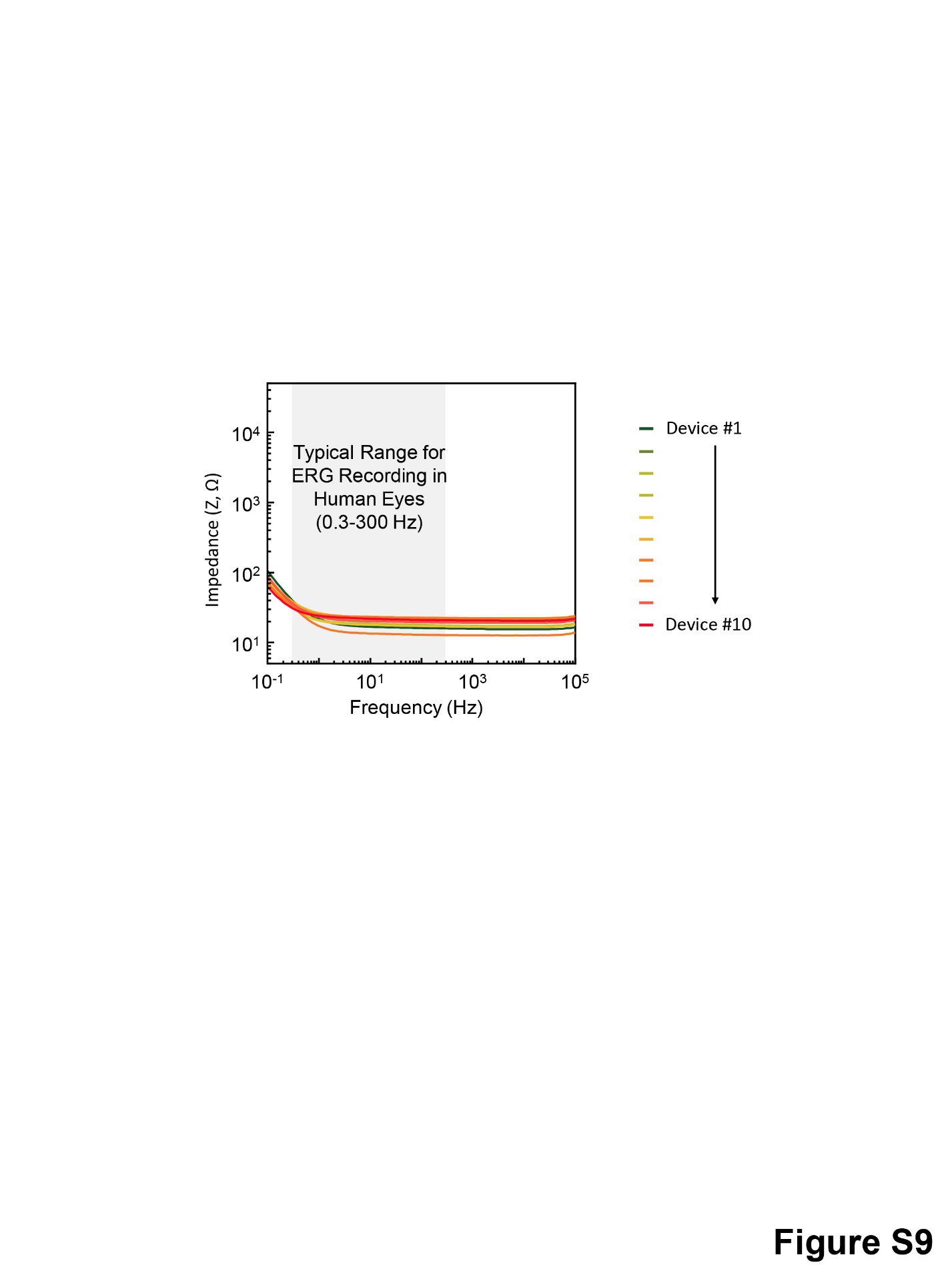
**

**Supplementary Figure S9. Device-to-device variation.** Electrochemical impedance of the randomly chosen corneal sensors (n = 10) as a function of frequency. The typical frequency range of ERG recordings in human eyes is highlighted in grey color.


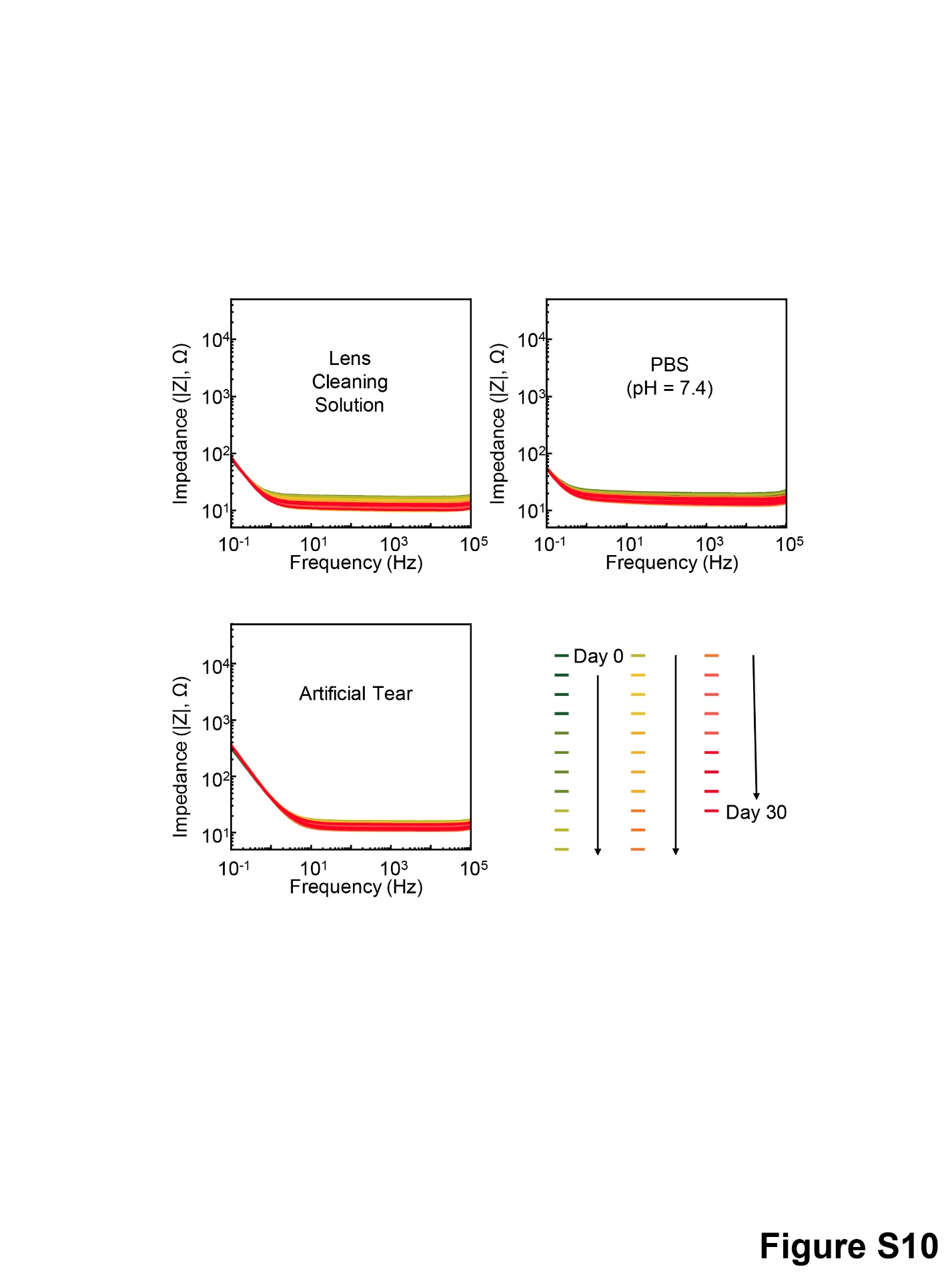


**Supplementary Figure S10. Long-term chemical stability of the corneal sensor in various aqueous media.** Electrochemical impedance of the corneal sensor immersing in a lens cleaning solution (top left graph), PBS (pH = 7.4; top right graph), and artificial tear (bottom left graph) for 30 days.


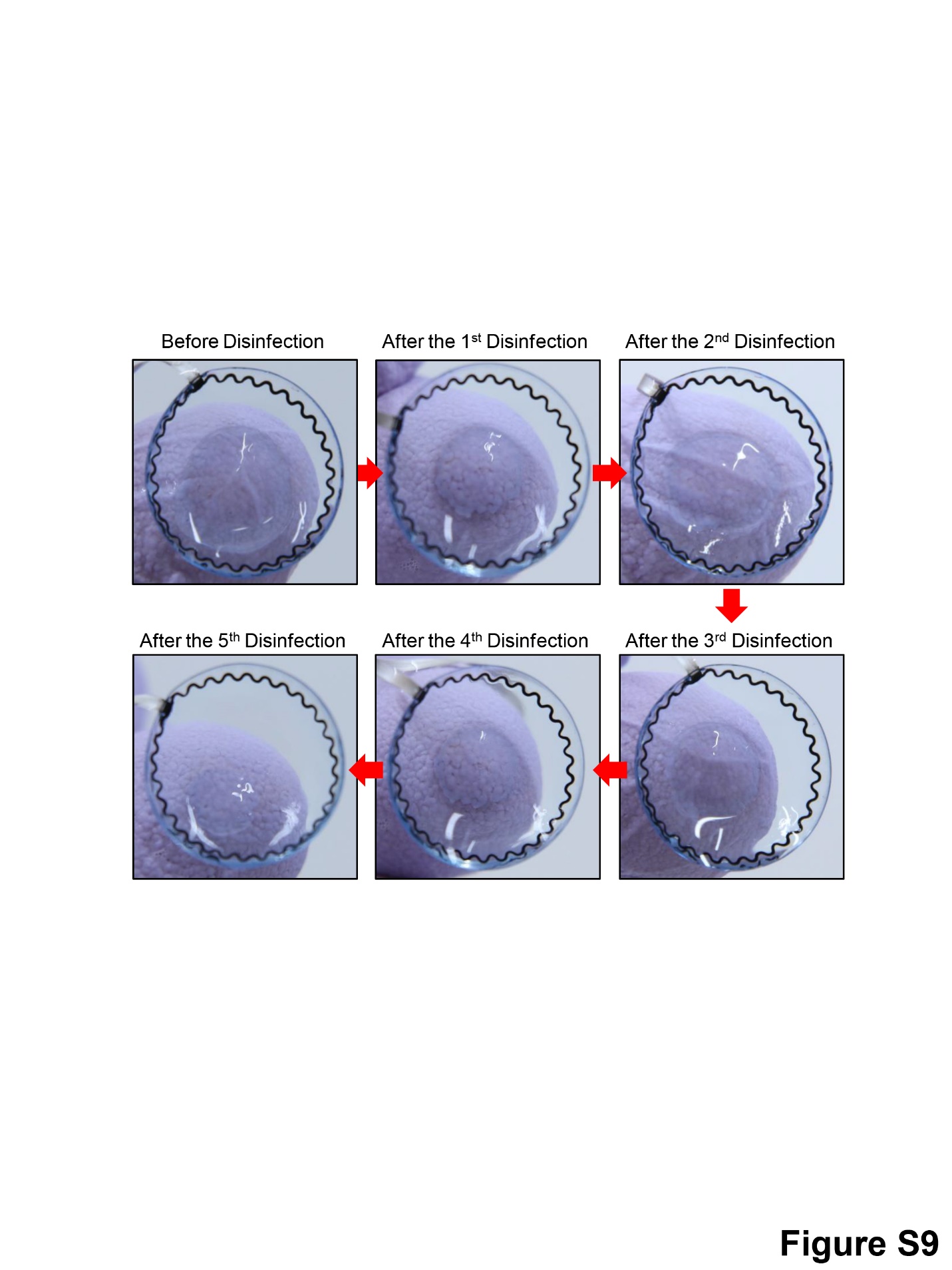


**Supplementary Figure S11. Stability under multiple cycles of disinfection process.** A series of photographs of the corneal sensor throughout the 5 cycles of disinfection process using a 3% H_2_O_2_ formula (ClearCare^®^, Alcon) over 12 hours each.


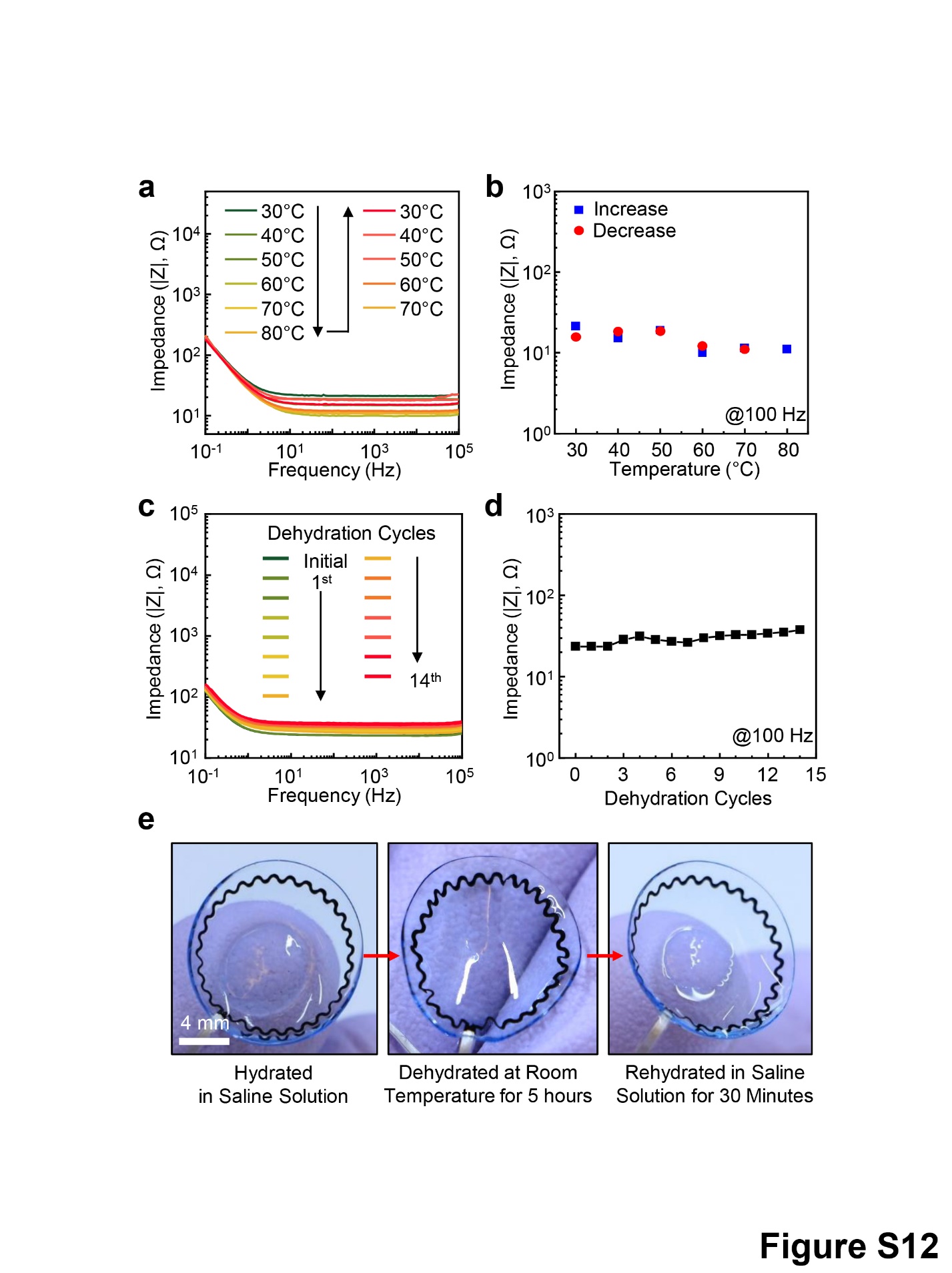


**Supplementary Figure S12.** **Electrochemical impedance against various conditions.** **a,** Electrochemical impedance of the corneal sensor against a temperature cycling between 30°C and 80°C. **b,** The corresponding results with the fixed frequency of 100 Hz. **c,** Electrochemical impedance of the corneal sensor against multiple dehydrations in ambient condition for at least 5 hours each. **d,** The corresponding results with the fixed frequency of 100 Hz. **e,** Optical images of the corneal sensor throughout a cycle of dehydration and rehydration.

**
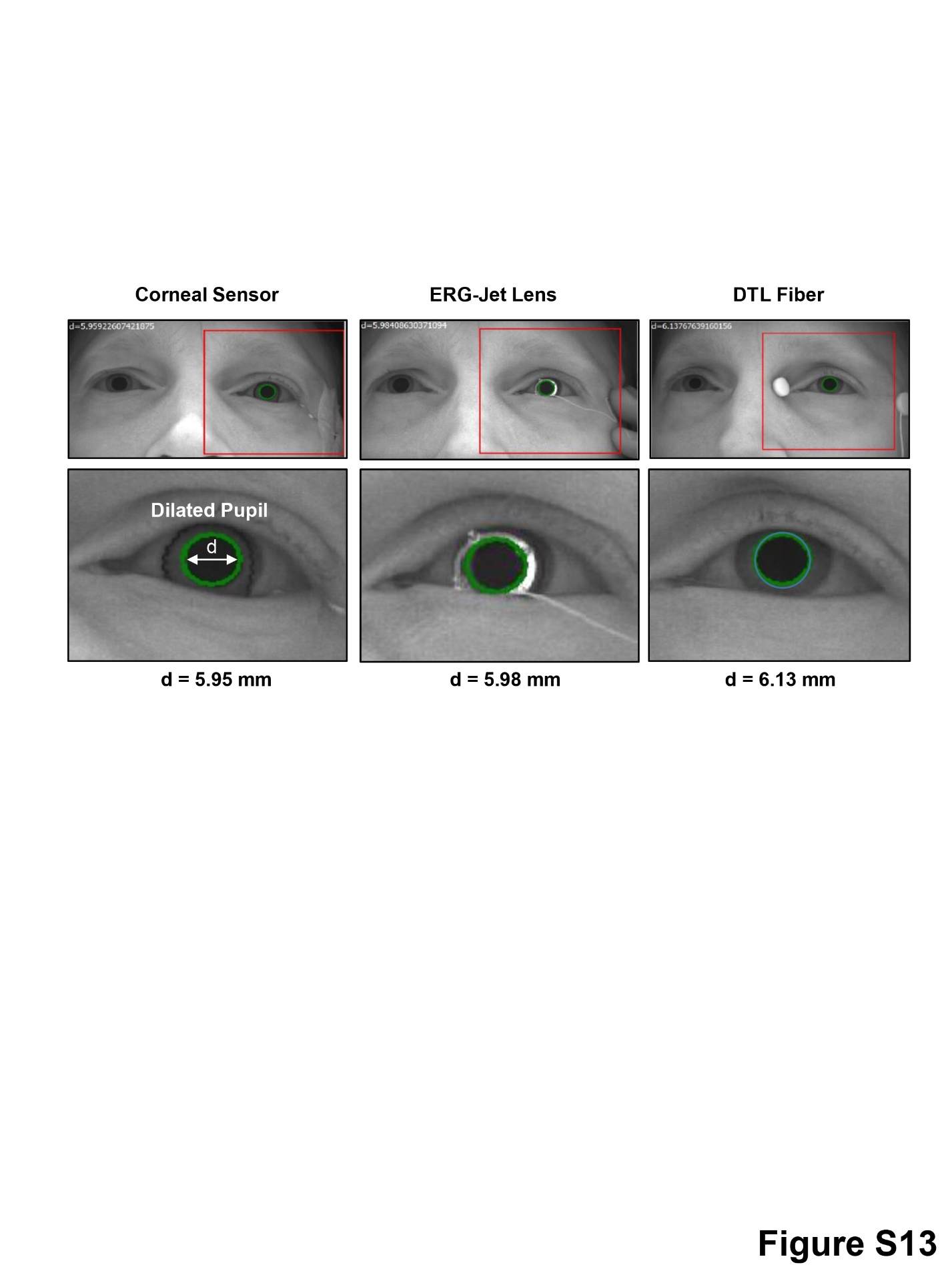
**

**Supplementary Figure S13. Automatic detection of fully dilated pupil during ERG recording.** Representative IR images of the left eye of the participant worn with the corneal sensor (left column) by comparisons with the ERG-Jet lens (middle column) and the DTL fiber (right column). The diameter of the fully dilated pupil is noted below each image.


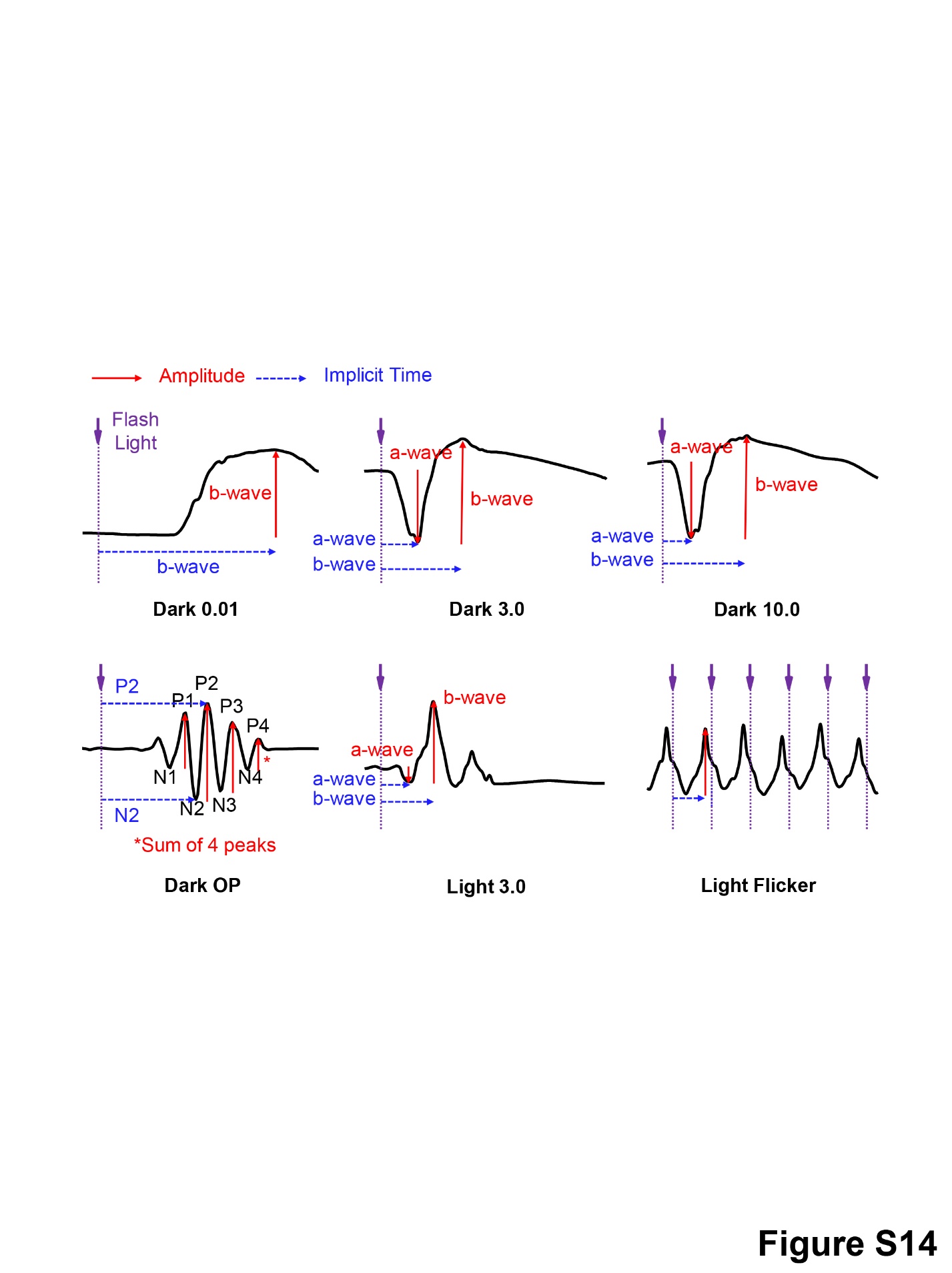


**Supplementary Figure S14. ISCEV standard** **full-field ERG signals.** The amplitudes (red lines) and implicit times (blue lines) of the a- and b-waves obtained from each ERG protocol are noted. Purple dotted lines indicate the moment of flashing light. In case of the Dark OP, the amplitude is considered as the sum of the 4 red arrows.

**
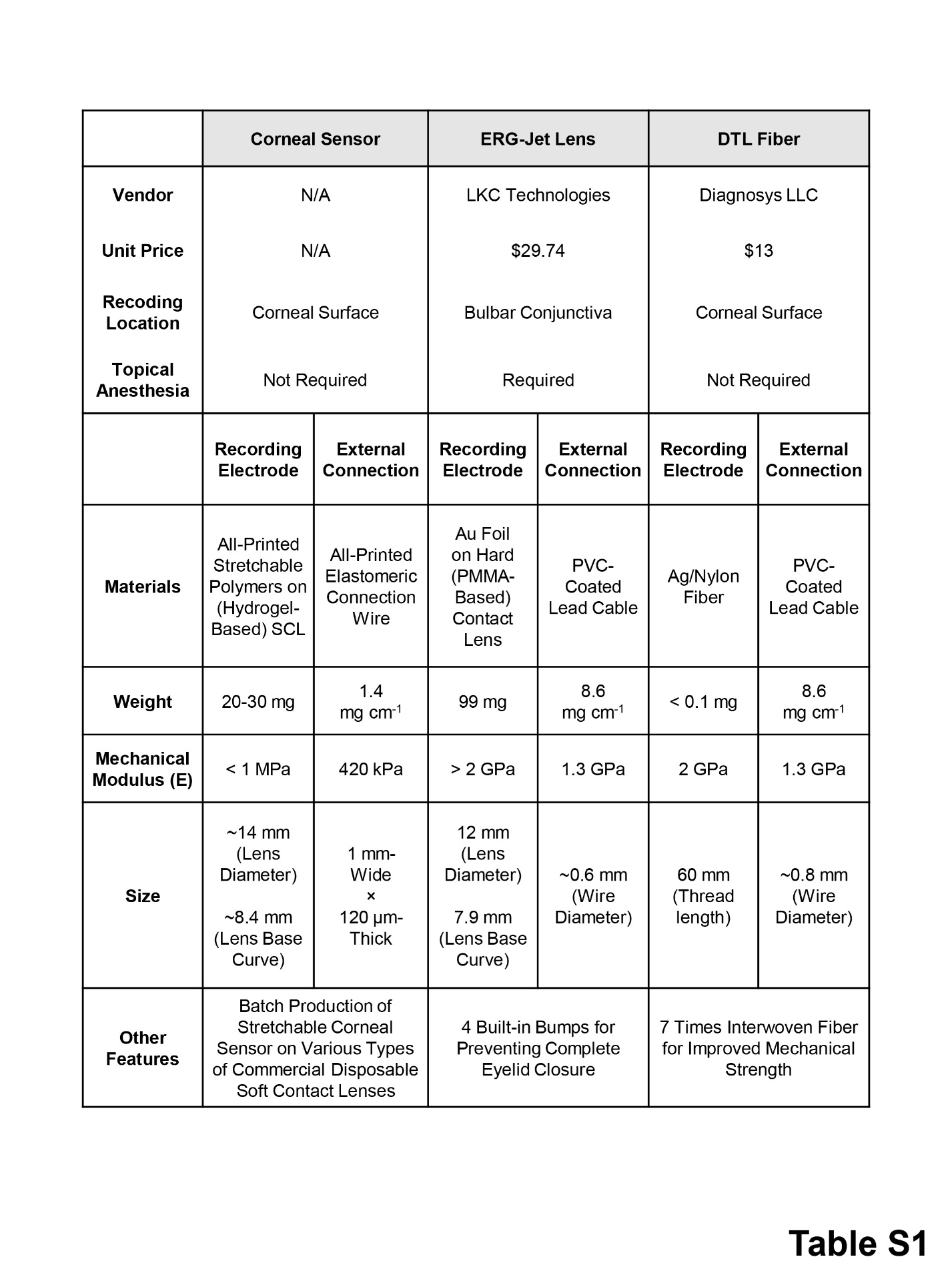
**

**Supplementary Table S1.** Comparisons of the technical specifications of the three different devices used in this study. Their current market prices are included as of May 2020.

**
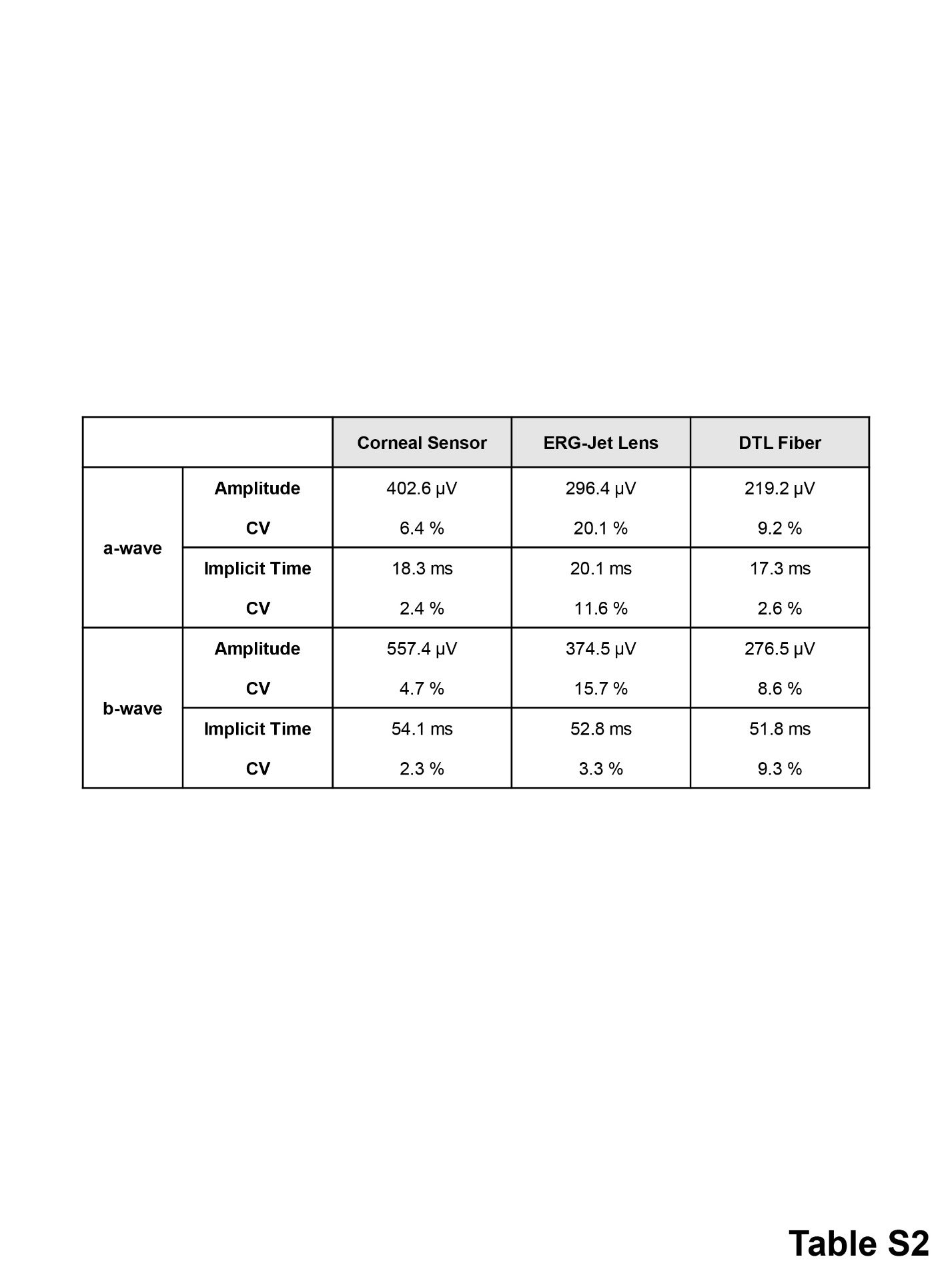
**

**Supplementary Table S2.** Comparisons of the average amplitudes and implicit times of the a- and b-waves extracted from full-field ERG signals acquired using the three different devices used in this study. The corresponding coefficient of variation (CV) for each amplitude and implicit time is noted.
