# Supplementary material for: All-printed stretchable corneal sensor on soft contact lenses for noninvasive and painless ocular electrodiagnosis
Source: Nat Commun. 2021 Mar 9;12:1544. doi: 10.1038/s41467-021-21916-8 (PMC7943761; doi:10.1038/s41467-021-21916-8)
Supplement: Supplementary file 3 — Description of Additional Supplementary Files [file 41467_2021_21916_MOESM3_ESM.pdf]

Description of Additional Supplementary Files

File Name: Supplementary Movie S1
Description: Automated batch production (10 units per batch) using a computer-controlled dispenser-printing tool.

File Name: Supplementary Movie S2
Description: Stretching of the corneal sensor.

File Name: Supplementary Movie S3
Description: Folding and scrubbing of the corneal sensor

File Name: Supplementary Movie S4
Description: The participant wearing the corneal sensor with natural blinking and eye movements.

File Name: Supplementary Movie S5
Description: Real-time IR imaging during ERG recordings by using the corneal sensor.

File Name: Supplementary Movie S6
Description: Real-time IR imaging during ERG recordings by using the ERG-Jet lens.
